# Effect of fruit on glucose control in diabetes mellitus: a meta-analysis of nineteen randomized controlled trials

**DOI:** 10.3389/fendo.2023.1174545

**Published:** 2023-05-05

**Authors:** Yu Ren, Shuang Sun, Yongwei Su, Chenfei Ying, Hua Luo

**Affiliations:** ^1^ Department of Pharmacy, Taizhou Hospital of Zhejiang Province affiliated to Wenzhou Medical University, Taizhou, Zhejiang, China; ^2^ Key Laboratory of Pathobiology, Ministry of Education, Nanomedicine and Translational Research Center, The Third Bethune Hospital of Jilin University, Changchun, Jilin, China; ^3^ Department of Orthopedic, The First Affiliated Hospital of Jinzhou Medical University, Jinzhou, Liaoning, China; ^4^ Department of Orthopedic, Taizhou Hospital of Zhejiang Province affiliated to Wenzhou Medical University, Taizhou, Zhejiang, China

**Keywords:** fruit, glucose, diabetes, glycosylated hemoglobin, meta-analaysis

## Abstract

**Objective:**

Diabetes mellitus is a worldwide health problem, and it remains unclarified whether fruit is beneficial in glycemic control. This study aimed to analyze evidence from randomized controlled trials evaluating the effect of fruit consumption on glucose control.

**Methods:**

We searched the PubMed, EMBASE, Ovid, Web of Science, and Cochrane Central Register of Controlled Trials databases from the respective database inception dates to December 30, 2022, to identify randomized controlled trials that evaluated the effects of fruit consumption on glucose control. Two researchers independently screened the studies in accordance with the inclusion and exclusion criteria, and performed the literature quality evaluation and data extraction. RevMan 5.4 software was used to perform the data analysis.

**Results:**

Nineteen randomized controlled trials with 888 participants were included. Fruit consumption significantly decreased the fasting blood glucose concentration (MD -8.38, 95% CI -12.34 to -4.43), but it showed no significant difference in the glycosylated hemoglobin (MD -0.17, 95% CI -0.51 to 0.17). Subgroup analyses further suggested that the consumption of both fresh and dried fruit decreased the fasting blood glucose concentration.

**Conclusions:**

Increasing the fruit intake reduced fasting blood glucose concentration. Therefore, we recommend that patients with diabetes eat more fruits while ensuring that their total energy intake remains unchanged.

## Introduction

According to the International Diabetes Federation Diabetes Atlas of 2021, there are 536.6 million adults living with diabetes worldwide (1). Complications arising from poor glycemic control are a major risk factor against healthy survival in patients with diabetes, and it is unclear whether glycemic control is influenced by fruit consumption. The Australian Diabetes, Obesity and Lifestyle Study of 7675 participants with an average age of 54 years showed that those who ate about two servings of fruit (including apples, bananas, and oranges) per day had a 36% lower risk of developing type II diabetes over the subsequent 5 years than those who ate less than half a serving of fruit per day (2). There is also a clear negative correlation between fruit intake and markers of insulin sensitivity, meaning that people who eat more fruit need to produce less insulin to lower their blood glucose concentration (2). This is important because abnormal metabolism in diabetes, hypertension, vascular sclerosis, obesity, and heart disease is strongly associated with hyperinsulinemia (3, 4). Furthermore, an unhealthy diet lacking fruits and vegetables increases the risks of cardiovascular disease and tumorigenesis (5).

Fresh fruits are rich in dietary fiber, organic acids, minerals, and antioxidants (e.g., vitamins, polyphenols), and contain small amounts of fat and calories, which helps regulate the composition and metabolic activity of intestinal microbes and reduce the incidence of complications in patients with diabetes (6). Polyphenols have a variety of important biological activities, including antiviral, antibacterial, anti-inflammatory, anticancer, and antioxidant. The most important of these activities is antioxidation, which converts free radicals into more stable free radicals to promote their scavenging ability, or reduces reactive oxygen species production by inhibiting mitochondrial oxidative stress to maintain cellular redox homeostasis (7). A 7-year prospective study of 500,000 Chinese adults showed a 17% reduction in mortality among patients with diabetes who consumed fruit on 3 or more days per week, as well as a 28% and 13% reduction in the risks of diabetic microvascular complications and macrovascular complications, respectively (8). Recent studies of patients with diabetes have shown that flavonoid-rich fruit intake is associated with lower glycosylated hemoglobin (HbA1c) and fasting blood glucose concentrations, and that increased flavonoid fruit intake reduces the risk of retinopathy by 30% (9).

In recent years, increasing attention has been paid to the impact of fresh fruit intake on type 2 diabetes. Fruit has a high sugar content and fruit consumption may affect the blood glucose management. Traditional beliefs have led patients with diabetes to eat only a limited variety of fruits with a low sugar content, such as cucumbers. However, recent studies have shown that fruit consumption has no significant effect on the fasting blood glucose concentration and the blood glucose concentration at 2 hours after meals compared with a control group (10). Furthermore, fruit intake reportedly helps control the HbA1c concentration (11). In addition, another study reported that fruits not only improve the postprandial concentrations of glucose and triglycerides, but also reduce inflammation and cardiovascular risk. Although many clinical studies have assessed the impact of fruits on the fasting blood glucose concentration, most were not large-scale, randomized, controlled trials (RCTs). Therefore, the present meta-analysis combined the previous research findings to explore the relationship between fruit intake and blood glucose control in patients with diabetes, to provide a reference for the consumption of fruits by patients with type 2 diabetes.

## Materials and methods

According to the PRISMA (Preferred Reporting Items for Systematic Reviews and Meta-Analyses) statement, this meta-analysis was performed in agreement (12). The protocol for this meta-analysis was registered on PROSPERO (Registration No: CRD42021237276).

### Inclusion criteria

(1) RCTs with human participants; (2) studies evaluating fruit intake and control of blood glucose concentration; (3) studies including patients diagnosed with diabetes or prediabetes; (4) studies with a follow-up of at least 1 month; (5) studies that assessed the BPG or HbA1c concentration; (6) studies that assessed the intake of fresh fruit, fruit juice, dry fruit, or fruit powder.

### Exclusion criteria

(1) Letters, case reports, reviews, observational studies, animal trials, or republished studies; (2) cohort studies; (3) studies that assessed the intake of fruit extract; (4) studies that included patients diagnosed with type 1 diabetes.

### Outcomes

The primary outcome was the difference in the blood glucose response to fruit consumption compared with control. The second outcome was the difference in the HbA1c concentration between a group that consumed fruit versus a control group. The serum lipid and lipoprotein concentrations were also analyzed.

### Search strategy

Two researcher searched the PubMed, Ovid, Web of Science, and Cochrane Central Register of Controlled Trials databases from their respective inception dates to December 30, 2022, using the keywords “(blood glucose or serum glucose or plasma glucose or glycemic or glycaemic or HbA1c or Hemoglobin A1c) and (fruit or juice) and (diabetes or diabetic or prediabetes or insulin resistance or impaired glucose tolerance) and (randomized controlled trial OR controlled clinical trial OR randomized OR randomly)”. We also searched the International Clinical Trials Registry Platform maintained by the World Health Organization to identify ongoing or unpublished eligible trials, and searched the reference lists of articles retrieved in the database search to identify related articles. No language restrictions were applied during the literature search.

### Study selection

After the removal of duplicates, two independent researchers (SS and YR) screened all titles and abstracts and determined the study eligibility in accordance with the inclusion and exclusion criteria. For studies considered eligible, the researchers obtained the full text and performed further screening. When the two researchers had differing opinions regarding the eligibility of a study for inclusion and could not reach a consensus, the senior researcher made the final decision after a group discussion.

### Data collection

Two researchers (HL and YR) used a standard data extraction form to independently extract all related data from the selected RCTs. When the RCTs had more than two groups and permitted multiple comparisons, we pooled only the data and information of interest reported in the original article. If a study included more than one intervention, we combined groups to create a single pair-wise comparison. The extracted data included the first author’s name, year of publication, type of study, sample size, interventions in the control and experimental groups, and follow-up duration. Disagreements were resolved by consensus.

### Assessment of risk of bias

Two researchers (YWS and YR) independently assessed the quality of all included RCTs based on the Cochrane risk-of-bias criteria (13).

### Data synthesis

The meta-analysis was performed using RevMan software (version 5.4; The Cochrane Collaboration). The heterogeneity was assessed by the Q test and I^2^ value. If heterogeneity was not present (P>0.1 and I^2^<50%), the data were combined with a fixed effect model. If heterogeneity was present (P<0.1 or I^2^ >50%), the random effects model was used. The mean difference (MD) and associated 95% confidence interval (CI) were used to assess outcomes, and P<0.05 was taken to indicate a significant difference. The possibility of small study effects was assessed qualitatively by visual estimations of funnel plots.

### Subgroup analyses

We performed several subgroup analyses to test interactions in accordance with the intake of fresh fruit or dry fruit.

### Sensitivity analyses

We performed sensitivity analyses by excluding the largest trial, excluding cluster-randomized or quasi-randomized trials, excluding trials with a high risk of bias, and using random effect models.

## Results

### Eligible studies and study characteristics

**Figure 1:**
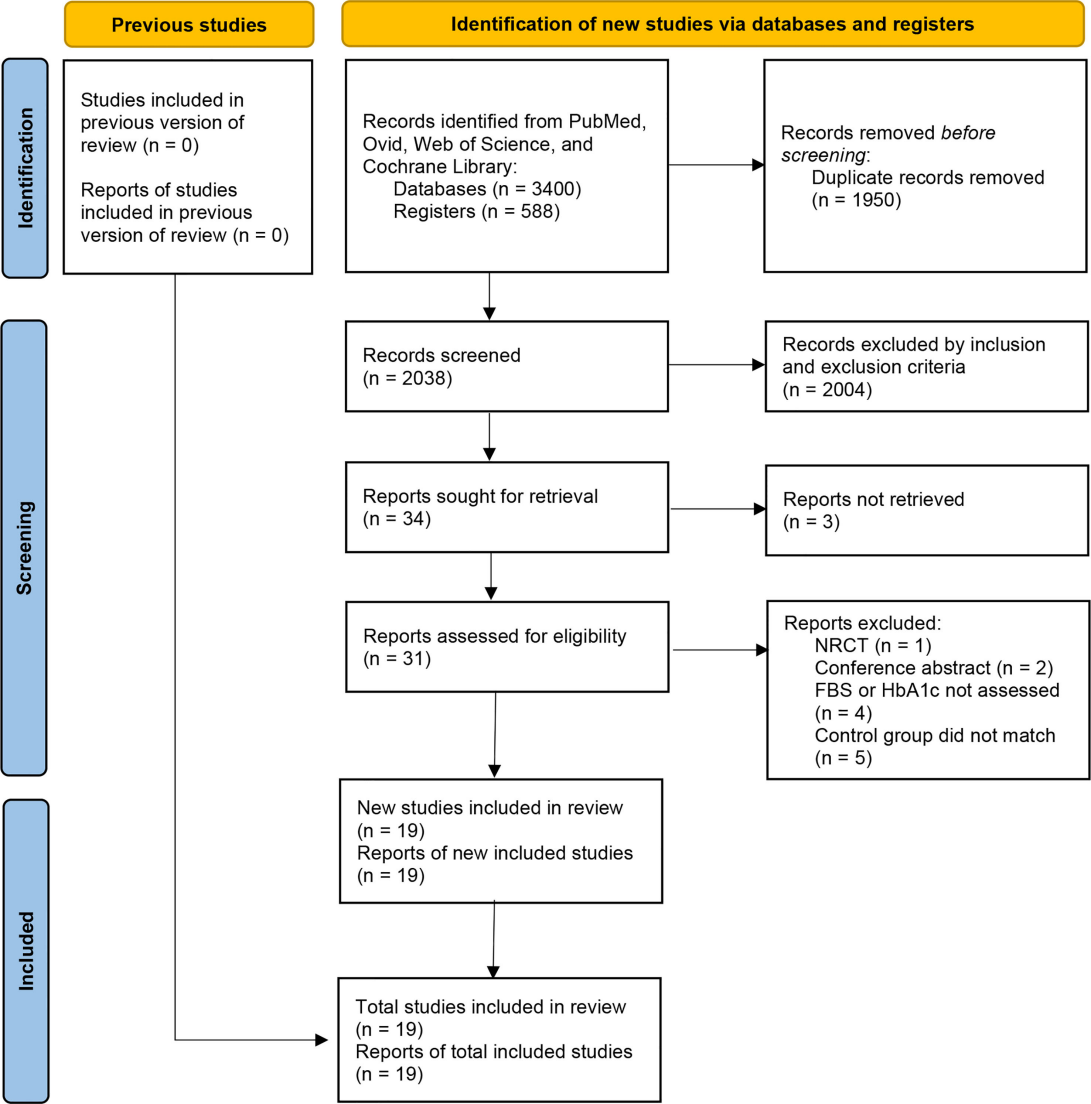
Flow diagram for search and selection of included studies.

**Figure 2:**
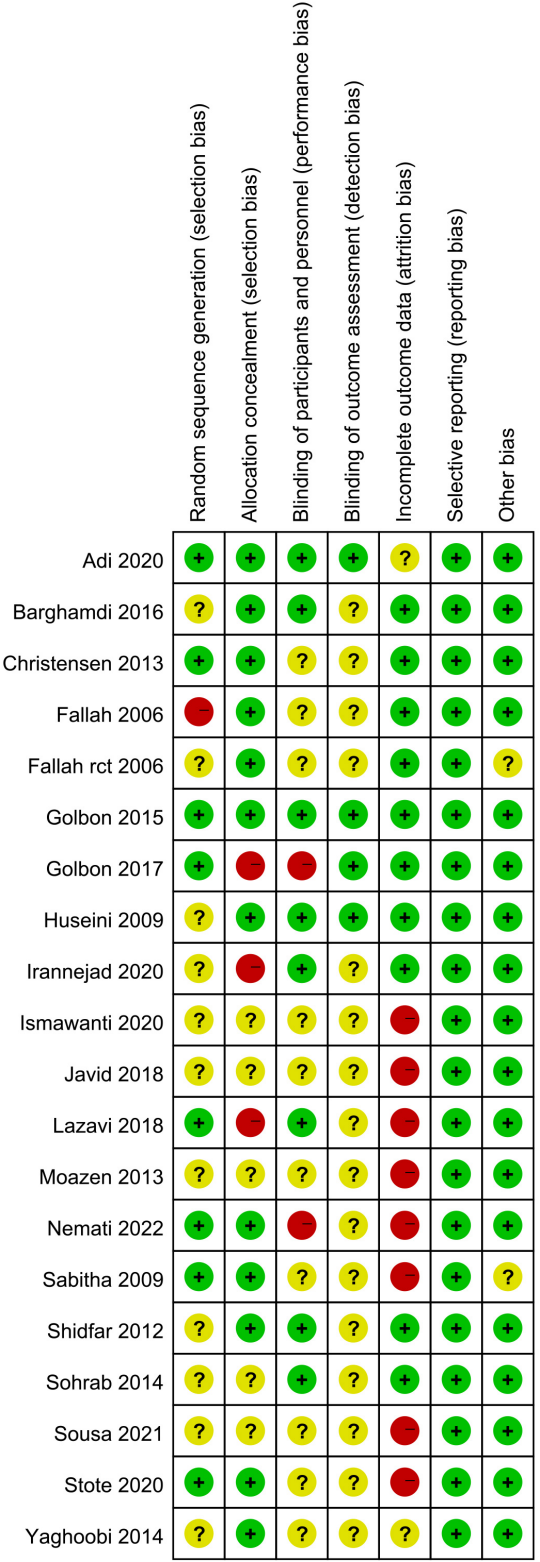
Risk of bias summary: review authors’ judgements about each risk of bias item for each included study.

We initially identified a total of 3988 articles, and finally included nineteen eligible RCTs with 888 participants in the meta-analysis (11, 14–31). The study flow chart is shown in **Figure 1**. A summary of the included RCTs is shown in **Table 1**. The risk of bias of each of the included RCTs is shown in **Figure 2**.

**Table 1:** Characteristics of included studies.

Study	No. of subjects		Country	Study design	Participants	Duration	Treatment group	Control group	Outcomes
	Fruit	Control							
Adi et al. (14)	17	13	India	RCT	T2DM	6 M	Syzygium 30 potency bid	Placebo	FBG, HbA1c
Barghamdi et al. (15)	35	35	Iran	RCT	T2DM	2 M	C. colocynthis capsule 125 mg qd	Placebo (Starch powder)	FBG, HbA1c
Fallah-1 (17)	22	22	Iran	RCT	T2DM	2 M	Citrullus colocynthis capsule 100 mg tid	Placebo (similar capsule)	FBG, HbA1c, Oxidative parameters
Fallah-2 (16)	25	25	Iran	RCT	T2DM	2 M	Citrullus colocynthis capsule 100 mg tid	Placebo (similar capsule)	FBG, HbA1c, lipid profile, SGOT, SGPT, ALK, BUN, serum creatinine
Golbon et al. (18)	22	22	Iran	RCT	T2DM	12 W	Pomegranate juice 250 ml qd	Placebo (similar color beverage)	FPS, HbA1c, insulin, oxidative stress, and advanced glycated end-products markers
Golbon et al. (19)	30	30	Iran	RCT	T2DM	12 W	Pomegranate juice 200 ml qd	No Treatment	FPS, Oxidized LDL and anti-oxidized LDL antibodies
Huseini et al. (20)	25	25	Iran	RCT	T2DM	2 M	Citrullus colocynthis capsules 100 mg tid	Placebo	FBG, HbA1c, lipid profile, aspartate transaminase, alanine transaminase, alkaline phosphatase, urea and creatinine
Irannejad.et al. (21)	36	36	Iran	RCT	T2DM	12 W	Dried Z. vulgaris 15 g bid	No Treatment	FBG, HbA1c, lipid profile, DBP, SBP, AST, and ALT
Ismawanti et al. (22)	25	13	Indonesia	RCT	T2DM	2 W	Red guavas 282.2 g or papaya 302.4 g bid	Placebo (mineral water)	FBG
Javid et al. (23)	9	12	Iran	RCT	T2DM	NA	Cranberry juice 200 ml qd	Placebo	FBG, HbA1c, lipid profile
Lazavi et al. (11)	21	21	Iran	RCT	T2DM	8 W	Barberry juice 200 ml qd	No Treatment	FBG, HbA1c, lipid profile
Moazen et al. (24)	19	17	Iran	RCT	T2DM	6 W	Dried strawberry powder 50 g qd	Placebo	FBG, HbA1c, malondialdehyde, C-reactive protein
Nemati et al. (25)	9	10	Iran	RCT	T2DM	8 W	Pomegranate juice 240 ml qd	Placebo (water)	FBG, Insulin, liver enzymes
Sabitha et al. (26)	17	16	India	RCT	T2DM	3 W	Noni fruit juice 15 ml bid	Placebo	FBG, lipid profile
Shidfar et al. (27)	29	29	Iran	RCT	T2DM	12 W	Cranberry juice 240 ml qd	Placebo(mineral water)	FBG, paraoxonase-1 activity, apoA-1, apoB
Sohrab et al. (28)	22	22	Iran	RCT	T2DM	12 W	Pomegranate juice 250 mL qd	Placebo (similar color beverage)	FBG, insulin, HOMA-IR, infl ammatory markers
Sousa et al. (29)	28	28	Brazil	RCT	T2DM	4 M	Yellow passion fruit 500 mg tid	Placebo	FBG, HbA1c
Stote et al. (30)	26	26	USA	RCT	T2DM	8 W	Freeze-dried blueberries 11 g bid	Placebo	FBG, HbA1c, lipid profile
Yaghoobi et al. (31)	28	28	Iran	RCT	T2DM	1 M	Colocynthis 100 mg tid	Placebo (similar capsule)	FBG, HbA1c, lipid profile

RCT, randomized controlled trial; T2DM, Type 2 Diabetes Mellitus; M, month; W, week; HbA1c, Hemoglobin A1c; FBG, fasting blood glucose.

### Primary outcome: effect of fruit on glycemic control

**Figure 3:**
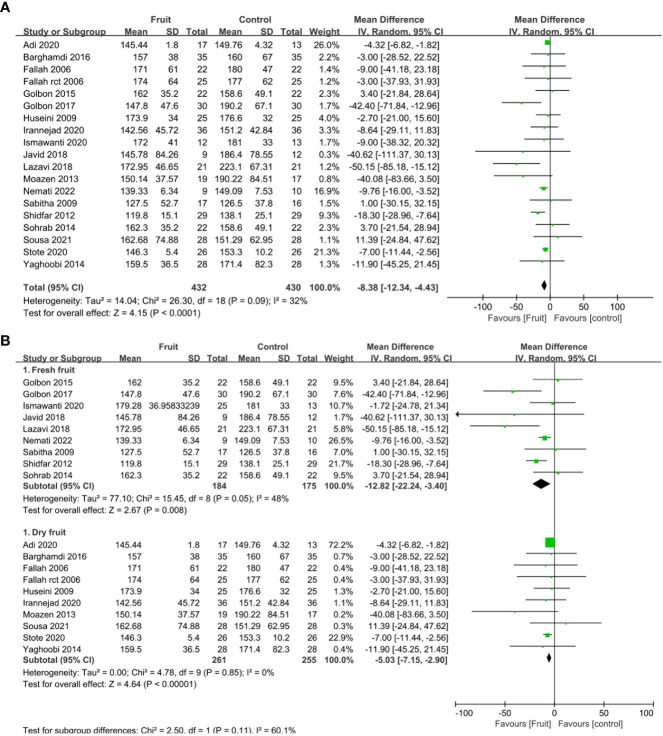
**(A)** Forest plot of comparison: fruit versus placebo; outcome: fasting blood glucose; **(B)** Forest plot of subgroup comparison: fruit versus placebo; outcome: fasting blood glucose.

All nineteen RCTs reported the fasting blood glucose concentration. Compared with the control group, fruit consumption reduced the fasting blood glucose concentration (MD -8.38, 95% CI -12.34 to -4.43, I^2 = ^32%; **Figure 3A**). Stratified subgroup analyses were conducted to determine whether the effect on the fasting blood glucose concentration differed for the intake of fresh fruit/fruit juice compared with dry fruit. Compared with control, the pooled result showed a significant reduction in fasting blood glucose concentration after the consumption of both fresh fruit (MD -12.82, 95% CI -22.24 to -3.4, I^2^ =^ ^48%; **Figure 3B1**) and dry fruit (MD -5.03, 95% CI -7.15 to -2.90, I^2^ =^ ^0%; **Figure 3B2**).

### Secondary outcomes

**Figure 4:**
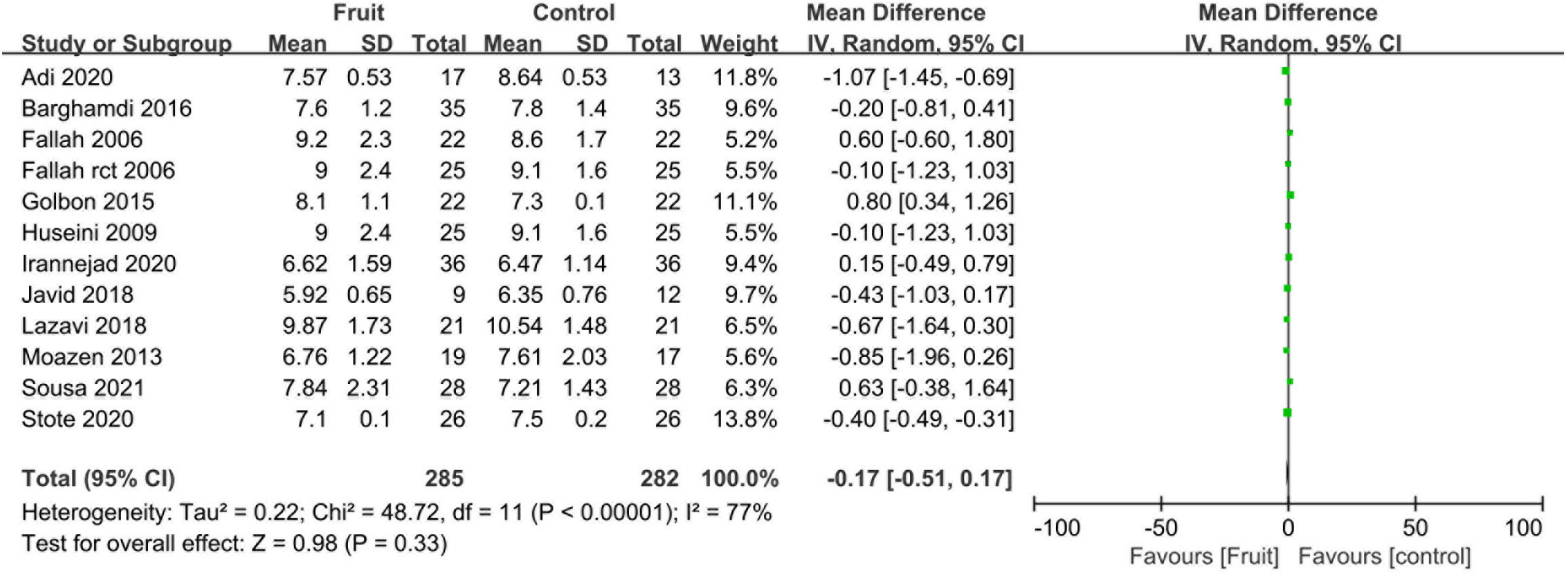
Forest plot of comparison: fruit versus placebo; outcome: Hb1Ac.

Pooled analysis showed no difference in the HbA1c concentration between the fruit consumption group and the control group (MD -0.17, 95% CI -0.51 to 0.17, I^2^ =^ ^77%; **Figure 4**).

## Discussion

Our meta-analysis showed that fruit consumption significantly reduced fasting blood glucose concentrations but had no significant effect on Hb1Ac concentrations in people with diabetes. We speculate that because the monosaccharides in fruits are mainly fructose-based, have a low blood glucose response, and are slow to absorb, the metabolism of fruit does not require the participation of insulin, and can be quickly cleared and transferred after reaching the liver (32). Another possible reason may be that some of the included fruits have anti-diabetic effects to some extent (14–17, 20, 21, 29, 31). Although the dry fruit had an increased sugar content compared with fresh fruit due to water loss, a subgroup analysis suggested that both dried and fresh fruit reduced blood glucose concentrations, and dried fruit has the characteristics of a long shelf-life and good transportability compared with fresh fruit.

Du et al. found that fruit consumption had a weak inverse, instead of positive, association with levels of blood glucose (8). Our results also suggest that eating fruit reduced the fasting blood glucose concentration. The available evidence suggests that the increase in the fruit intake should be accompanied by a corresponding decrease in the carbohydrate intake, ensuring that the total energy intake remained unchanged (6). The energy produced per 100 grams of fresh fruit is about 21–282 kcal (33). Our experience is that patients with stable blood glucose control who eat 200–250 g of fresh fruit per day need to reduce their daily intake of staple food by 25 g to avoid exceeding their total daily energy intake limit. Increasing the fruit intake is limited to patients with diabetes with stable glycemic control, while patients with diabetes with unstable blood glucose concentrations need to be cautious when increasing their fruit intake. A good method is to measure the urine glucose concentration at 2 hours after eating fruit. If the urine sugar increases, the patient needs to reduce the amount of fruit eaten; if the urine glucose is still high after reducing the fruit consumption, then the amount of staple food eaten needs to be appropriately reduced. In short, fruits should be considered as a part of a diet that has been comprehensively considered to achieve the purpose of not only supplementing nutrition but also controlling diabetes.

Diabetes is currently one of the main conditions that leads to a variety of macrovascular and microvascular complications (34). In severe cases, diabetes lead to disability and is even life-threatening. Although fruit is an important part of the daily diet, the high sugar content of most fruits has led to the conventional belief that fruit consumption may exacerbate diabetes, and thus the fruit intake of patients with diabetes is often severely restricted (35). However, fruits provide a lot of nutrients such as vitamins, fruit acids, minerals, and antioxidants, which help prevent the development and progression of many diseases; therefore, diabetes guidelines in the United States recommend eating more fruits (36). Antioxidants in fruits prevent cell damage by inhibiting lipid peroxidation and having lipotoxic effects (22). Furthermore, fruits may contain resistant starches and oligosaccharides that cannot be digested in the stomach and need to enter the colon for bacterial fermentation (37), which can stimulate the growth and/or activity of one or more beneficial bacteria, thereby improving human health (38). An 8-year follow-up study showed that diabetic retinopathy was significantly reduced in the group that consumed 253 g of fruit daily compared with the group that consumed 23 g of fruit daily (39). Increasing the fruit intake also reduces the incidences of stroke, coronary heart disease, and cancer (40–42). Other studies have shown beneficial effects of various fruits. For example, eating fresh grapefruit before meals significantly reduces weight while improving insulin resistance (43), polyphenols in strawberry fruits improve insulin sensitivity (44), and carambola juice may lower the blood glucose concentration and improve liver function in mice with diabetes (45).

Although studies have shown that the sugar load is higher after fruit juice is changed, the blood glucose concentration is easily increased by fruit juice consumption, which is not conducive to blood glucose control and causes vitamin and fiber loss, decreased satiety, and increased total energy; therefore, fruit juice is not recommended for patients with diabetes (46). However, Weisel et al. reported that fruit juice resulted in a decrease in oxidative DNA damage, an increase in reduced glutathione and glutathione status, a return to break-in levels during the subsequent elution period, and a significant reduction in oxidative damage in healthy proband cells in the juice (47). Other studies have shown that watermelon juice exerts antidiabetic effects in experimental animal models of diabetes by modulating glucose transporters, anti-inflammatory activity, antioxidant defense systems, and inhibiting multiple pathways such as α-glucosidase and α-amylase (48). Furthermore, the homeostasis of the gut flora is regulated by the ingestion of bioactive compounds found in citrus fruits and orange juice, such as hesperidin and naringenin (49).

To our knowledge, this is the first study to systematically review the potential effects of fruit consumption on glycemic control in patients with diabetes. The relatively large number of pooled participants achieved a greater statistical power than a single RCT. However, this study has some limitations. First, although this meta-analysis focused on studies evaluating the benefits of fruit consumption on human mechanisms, most of the included studies were assessed in Iran, which may increase the bias in the result. Different fruits have different sugar contents, and the types of fruits eaten varies between countries and regions. The sugar content can even vary between the same fruits with different levels of ripeness. Second, most of the fruits included in this meta-analysis have a certain medicinal value, which warrants further research. Third, most guidelines recommend a diet with a high intake of fiber-rich food, including fruit. This is based on the many positive effects of fruit on human health. However, some health professionals are concerned that fruit intake has a negative impact on glycemic control and therefore recommend restricting the fruit intake. We found no studies addressing this important clinical question (6). Fourth, the I^2^ value is the percentage of total variation across studies due to heterogeneity (between-study variability) rather than chance, with higher values indicating higher levels of heterogeneity between studies and higher inconsistency of results. As there was a high heterogeneity in the studies that assessed the Hb1Ac concentration, this conclusion needs to be further demonstrated by higher quality studies.

## Conclusions

Increasing the fruit intake reduced the fasting blood glucose concentration of patients with diabetes. Therefore, we still recommend that patients with diabetes eat more fruits while ensuring that their total energy intake remains unchanged. We concluded that moderate amounts of practical fruit products reduce blood glucose concentration, but the specific amounts of daily practical fruits need to be investigated in multicenter RCTs.

## Author contributions

YR and SS performed literature review. YR and HL collected data, performed data analysis, interpreted results, and wrote the first draft of the manuscript. YS and YR assessed risk of bias, reviewed the protocol, screened articles, and reviewed the results and manuscript. CY contributed to the systematic review protocol and critically reviewed the results and manuscript. HL contributed to the protocol development and reviewed the manuscript. YR, SS, YS, CY, and HL critically revised successive drafts of the manuscript and approved the final version. HL has affirmed that this manuscript is an honest, accurate, and transparent account of the study being reported. that no important aspects of the study have been omitted. and that any discrepancies from the study as planned (and, if relevant, registered) have been explained. HL is the guarantor of this work and, as such, had full access to all the data in the study and takes responsibility for the integrity of the data and the accuracy of the data analysis. All authors contributed to the article and approved the submitted version.
